# Supplementary material for: Trypanosomatid parasite dynamically changes the transcriptome during infection and modifies honey bee physiology
Source: Commun Biol. 2020 Jan 31;3:51. doi: 10.1038/s42003-020-0775-x (PMC6994608; doi:10.1038/s42003-020-0775-x)
Supplement: Supplementary file 1 — Supplementary Information [file 42003_2020_775_MOESM1_ESM.pdf]

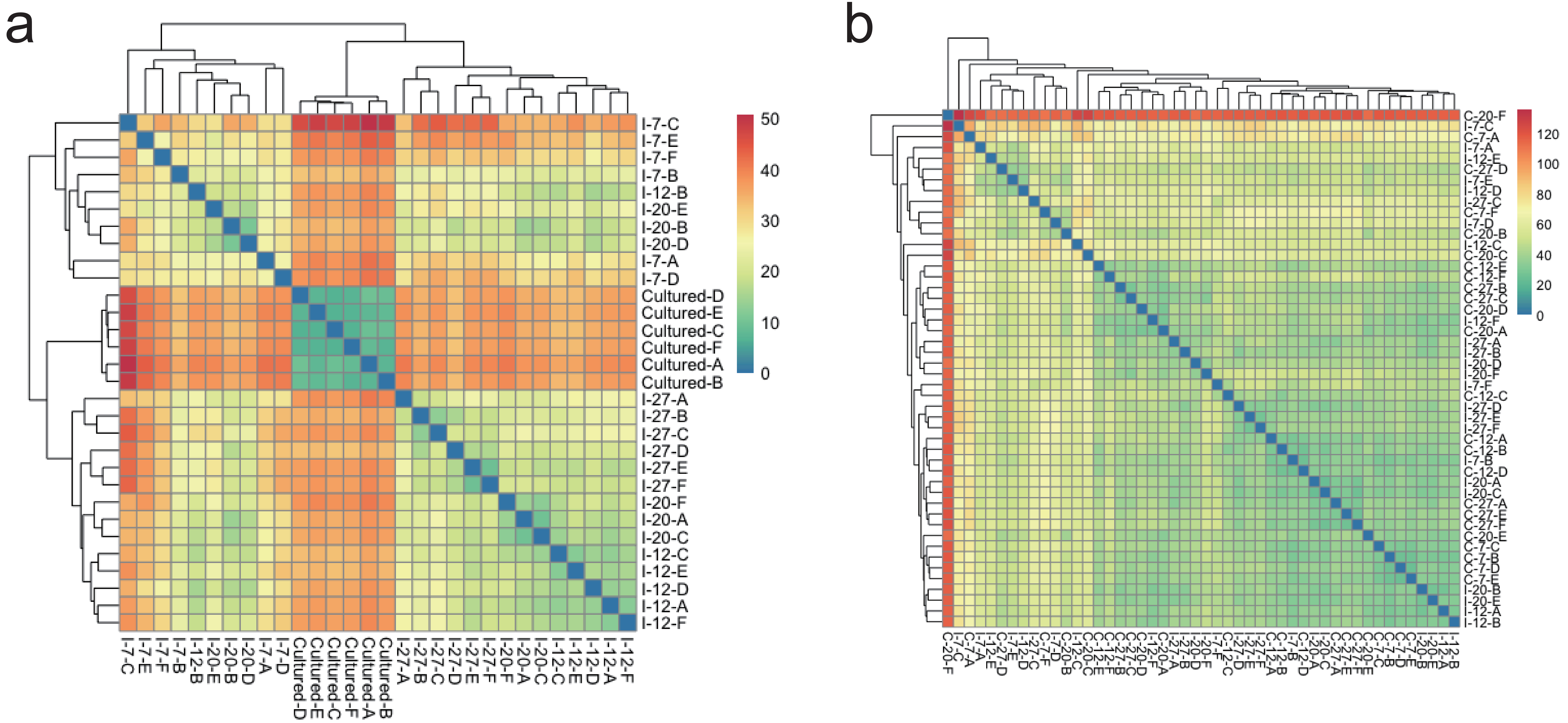
a
I-7-C
I-7-E
I-7-F
I-7-B
I-12-B
I-20-E
I-20-B
I-20-D
I-7-A
I-7-D
Cultured-D
Cultured-E
Cultured-C
Cultured-F
Cultured-A
Cultured-B
I-27-A
I-27-B
I-27-C
I-27-D
I-27-E
I-27-F
I-20-F
I-20-A
I-20-C
I-12-C
I-12-E
I-12-D
I-12-A
I-12-F
50
40
30
20
10
0
b
C-20-F
I-7-C
C-7-A
I-7-A
I-12-E
C-27-D
I-7-E
I-12-D
I-27-C
C-7-F
I-7-D
C-20-B
I-12-C
C-20-C
C-12-E
C-12-F
C-27-B
C-27-C
C-20-D
I-12-F
C-20-A
I-27-A
I-27-B
I-20-D
I-20-F
I-7-F
C-12-C
I-27-D
I-27-E
I-27-F
C-12-A
C-12-B
I-7-B
C-12-D
I-20-A
I-20-C
C-27-A
C-27-E
C-27-F
C-20-E
C-7-C
C-7-B
C-7-D
C-7-E
I-20-B
I-20-E
I-12-A
I-12-B
120
100
80
60
40
20
0

Supplementary Figure 1. Variation in *L. passim* and honey bee transcriptomic profiles

Heat maps to show the results of hierarchical analysis of 30 *L. passim* (a) and 48 honey bee (b) transcriptomes using Pearson correlation. The levels of difference between samples are indicated by different colors.

Supplementary Methods

Hierarchical analysis of the RNA-seq samples was conducted by DEseq2 (Version 1.22.1) and the Pearson correlation-based heat map was visualized using the gplots pheatmap.2 function to show distances between the RNA-seq (transcriptome) samples[1].

Supplementary References

1. Love, M. I., Huber, W. & Anders, S. Moderated estimation of fold change and dispersion for RNA-seq data with DESeq2. *Genome Biol.* **15,** 550 (2014).
