# Supplementary material for: Trypanosomatid parasite dynamically changes the transcriptome during infection and modifies honey bee physiology
Source: Commun Biol. 2020 Jan 31;3:51. doi: 10.1038/s42003-020-0775-x (PMC6994608; doi:10.1038/s42003-020-0775-x)
Supplement: Supplementary file 11 — Description of Additional Supplementary Items [file 42003_2020_775_MOESM11_ESM.docx]

Description of additional supplementary items

Supplementary Data 1

Mapping rates of 54 RNA-seq reads to either *A. mellifera* or *L. passim* genome

Supplementary Data 2

Lists of *L. passim* genes up- or down-regulated at 7, 12, 20, and 27 days after the infection as well as GO terms enriched with each gene list

Supplementary Data 3

*L. passim* genes continuously up-regulated throughout the infection and the enriched GO terms

Supplementary Data 4

*L. passim* genes continuously down-regulated throughout the infection and the enriched GO terms

Supplementary Data 5

*L. passim* genes up-regulated at 12-27 days after the infection

Supplementary Data 6

*L. passim* genes up-regulated at 12-27 days after the infection and the enriched GO terms

Supplementary Data 7

Lists of *L. passim* genes sequentially up- or down-regulated at 7-27 days after the infection as well as GO terms enriched with each gene list

Supplementary Data 8

Lists of *A. mellifera* genes up- or down-regulated at 7, 12, 20, and 27 days after the infection of *L. passim* as well as GO terms enriched with each gene list

Supplementary Data 9

Source data for graphs presented in Figure 1 and 4.
