# Supplementary material for: Trypanosomatid parasite dynamically changes the transcriptome during infection and modifies honey bee physiology
Source: Commun Biol. 2020 Jan 31;3:51. doi: 10.1038/s42003-020-0775-x (PMC6994608; doi:10.1038/s42003-020-0775-x)
Supplement: Supplementary file 13 — Reporting Summary [file 42003_2020_775_MOESM13_ESM.pdf]

nature research

Corresponding author(s): Tatsuhiko Kadowaki

Last updated by author(s): Jan 5, 2020

Reporting Summary

Nature Research wishes to improve the reproducibility of the work that we publish. This form provides structure for consistency and transparency in reporting. For further information on Nature Research policies, see Authors & Referees and the Editorial Policy Checklist.

Statistics

For all statistical analyses, confirm that the following items are present in the figure legend, table legend, main text, or Methods section.

n/a	Confirmed	
☐	☒	The exact sample size (n) for each experimental group/condition, given as a discrete number and unit of measurement
☐	☒	A statement on whether measurements were taken from distinct samples or whether the same sample was measured repeatedly
☐	☒	The statistical test(s) used AND whether they are one- or two-sided<br>*Only common tests should be described solely by name; describe more complex techniques in the Methods section.*
☒	☐	A description of all covariates tested
☒	☐	A description of any assumptions or corrections, such as tests of normality and adjustment for multiple comparisons
☐	☒	A full description of the statistical parameters including central tendency (e.g. means) or other basic estimates (e.g. regression coefficient) AND variation (e.g. standard deviation) or associated estimates of uncertainty (e.g. confidence intervals)
☐	☒	For null hypothesis testing, the test statistic (e.g. F, t, r) with confidence intervals, effect sizes, degrees of freedom and P value noted<br>*Give P values as exact values whenever suitable.*
☒	☐	For Bayesian analysis, information on the choice of priors and Markov chain Monte Carlo settings
☐	☒	For hierarchical and complex designs, identification of the appropriate level for tests and full reporting of outcomes
☒	☐	Estimates of effect sizes (e.g. Cohen's d, Pearson's r), indicating how they were calculated

Our web collection on statistics for biologists contains articles on many of the points above.

Software and code

Policy information about availability of computer code

Data collection	OmicsBox (1.2.4), HISAT2 (version 2.1.0), Tophat (version 2.1.0), cufflinks (version 2.2.1), cuffmerge, HTSeq-count.
Data analysis	OmicsBox (1.2.4), RStudio (1.1.383), edgeR (3.24.2), DEseq2 (1.22.1).

For manuscripts utilizing custom algorithms or software that are central to the research but not yet described in published literature, software must be made available to editors/reviewers. We strongly encourage code deposition in a community repository (e.g. GitHub). See the Nature Research guidelines for submitting code & software for further information.

Data

Policy information about availability of data

All manuscripts must include a data availability statement. This statement should provide the following information, where applicable:

- Accession codes, unique identifiers, or web links for publicly available datasets
- A list of figures that have associated raw data
- A description of any restrictions on data availability

PRJNA587465

Field-specific reporting

Please select the one below that is the best fit for your research. If you are not sure, read the appropriate sections before making your selection.

☒ Life sciences ☐ Behavioural & social sciences ☐ Ecological, evolutionary & environmental sciences

For a reference copy of the document with all sections, see nature.com/documents/nr-reporting-summary-flat.pdf

Life sciences study design

All studies must disclose on these points even when the disclosure is negative.

Sample size	For the RNA-seq analyses, 6 samples were used at each time point/treatment. To test the degree of L. passim infection in honey bee, 10 samples were analyzed at each time point. To characterize the effect of L. passim infection on the survival of honey bee, 288-330 samples were analyzed in total. For testing the bacterial abundance, 8 samples were used for each time point/treatment group. For analyzing honey bee Vg mRNA, 13 parasite-infected and 14 control (uninfected) bees were used.
Data exclusions	All of the data were used for the analysis.
Replication	The experiment to test the effect of L. passim infection on the survival of honey bee was repeated three times. The experiment to analyze honey bee Vg mRNA was repeated twice.
Randomization	All of the honey bees were randomly sampled from either hive or cage.
Blinding	We did not employ blinding in this study.

Reporting for specific materials, systems and methods

We require information from authors about some types of materials, experimental systems and methods used in many studies. Here, indicate whether each material, system or method listed is relevant to your study. If you are not sure if a list item applies to your research, read the appropriate section before selecting a response.

Materials & experimental systems

n/a	Involved in the study	
☒	☐	Antibodies
☐	☒	Eukaryotic cell lines
☒	☐	Palaeontology
☐	☒	Animals and other organisms
☒	☐	Human research participants
☒	☐	Clinical data

Methods

n/a	Involved in the study	
☒	☐	ChIP-seq
☒	☐	Flow cytometry
☒	☐	MRI-based neuroimaging

Eukaryotic cell lines

Policy information about cell lines

Cell line source(s)	Lotmaria passim strain SF (ATCC PRA-403) was purchased from ATCC.
Authentication	The L. passim strain was authenticated by ATCC.
Mycoplasma contamination	Cell line was not tested for mycoplasma contamination
Commonly misidentified lines (See ICLAC register)	None of them was used in this study.

Animals and other organisms

Policy information about studies involving animals; ARRIVE guidelines recommended for reporting animal research

Laboratory animals	This study did not use laboratory animals.
Wild animals	Honey bee (Apis mellifera) hives were obtained from a bee keeper in Suzhou, Jiangsu Province, China. Our hives were maintained at Xi'an Jiaotong-Liverpool University. Newly emerged and 2 days old honey bees were used for the experiments. The honey bees were returned to the original hives after the parasite infection, and then collected on day 7, 12, 20 and 27. Honey bees were sacrificed by forceps and all of the honey bee samples were frozen at the end of experiment.
Field-collected samples	This study did not use wild animals.
Ethics oversight	No ethical approval/guidance was required for this study since vertebrate and the infecting agents were not used.

Note that full information on the approval of the study protocol must also be provided in the manuscript.
